# A Latent Profile Analysis of Affective Triggers for Risky and Impulsive Behavior

**DOI:** 10.3389/fpsyg.2018.02651

**Published:** 2019-01-04

**Authors:** Emily Kemp, Naomi Sadeh, Arielle Baskin-Sommers

**Affiliations:** ^1^Department of Psychiatry, Yale University, New Haven, CT, United States; ^2^Department of Psychological and Brain Sciences, University of Delaware, Newark, DE, United States; ^3^Department of Psychology, Yale University, New Haven, CT, United States

**Keywords:** risk-taking, impulsive, affective triggers, latent profile analysis, self-report questionnaire

## Abstract

Common theoretical models of risky and impulsive behaviors suggest that individuals engage in risky behavior to avoid negative affect or enhance positive affect. However, little research has been done to identify person-centered affective profiles of risky and impulsive behavior, and delineate the individual differences across these profiles. The present study used the *Risky, Impulsive, and Self-destructive Behavior Questionnaire* in community (*N* = 439) and incarcerated (*N* = 262) samples to examine latent affect profiles for risky and impulsive behavior. Four affective profiles emerged: low avoidance and low approach, average avoidance and average approach, high avoidance, and high approach. Conditional probability correlations revealed meaningful differences across these profiles in psychiatric symptomatology, personality characteristics, and behavior. Consideration of affective triggers provides an important framework for dissociating the underlying reasons why individuals engage in risky behavior.

## Introduction

Risky and impulsive behavior encompasses a range of acts from speeding to substance misuse to aggression. Collectively these behaviors exact staggering costs by increasing the likelihood of premature death, long-term disability, and poor mental health outcomes (Moffitt et al., 2011; Centers for Disease Control and Prevention, 2014). Additionally, engagement in risky and impulsive behaviors, whether those behaviors are inherently criminal or not, increases the likelihood of justice system involvement (Chamorro et al., 2012; Bechtold et al., 2014; DeLisi and Vaughn, 2014; Mestre-Bach et al., 2018). The enormity of the problems risky and impulsive behavior brings to the individual and society at-large underscores the importance of identifying the factors that spur on these behaviors.

Engagement in risky and impulsive behaviors emerges in a wide variety of circumstances, but especially when individuals are in negative mood states or are influenced by immediate temptations (Loewenstein et al., 2001; Weiss et al., 2012). Accordingly, common theoretical models of risky and impulsive behavior identify two primary triggers: (1) to reduce or relieve negative affective states, such as extreme distress, sadness, or anger (i.e., “avoid” trigger; Whiteside et al., 2005; Leyro et al., 2010; Nock, 2010), and (2) to increase positive affective states, such as pleasurable or thrilling experiences (i.e., “approach” trigger; Horvath and Zuckerman, 1993; Zuckerman and Kuhlman, 2000).

On the one hand, states of negative affect can serve as triggers for risky and impulsive behavior. For example, exposure to drug-associated cues and contexts increase susceptibility to relapse and substance addicted individuals are highly vulnerable to relapse during periods of withdrawal-induced negative affect (Baker et al., 2004). Over valuing short-term relief over long-term goals is associated with suicidal behavior and this behavior is elevated in Veterans who are coping with deployment-related stressors (Tanielian, 2009). And, insensitivity to punishment and high stress levels increase the likelihood of engaging in risky sexual behavior, substance use, and other adverse health behaviors in college students (Nelson et al., 2008).

On the other hand, seeking positive affect may trigger risky and impulsive behavior. Among college students, positive affect increases the likelihood of illegal drug use and risky sexual behavior over the course of college (Zapolski et al., 2009). In treatment-seeking pathological gamblers, positive mood is a trigger for resuming gambling (Holub et al., 2005). Adolescents and adults who engage in public riots often report recreation, “fun,” and adventure seeking as primary motivators for their behavior (Leonard, 2010; Willmott and Ioannou, 2017). Finally, positive urgency predicts some forms of violence, such as intimate partner violence (Derefinko et al., 2011).

Notably, these two affective triggers are not mutually exclusive. While for some individuals, negative affect triggers risky and impulsive behavior, for other individuals, positive affect may be the trigger, for others both negative and positive affect may be the trigger, and for some, risky and impulsive behavior may occur without an affective trigger. Understanding the diversity in affective triggers for risky and impulsive behavior is essential for accurately identifying individuals who engage in these behaviors, and eventually intervening based on the underlying etiological and reinforcement mechanisms subserving their behavior. However, little research has been done to actually define affective profiles in a range of risky behaviors and delineate the individual differences across these profiles.

The goal of the present study was to explore dissociable profiles of risky and impulsive behavior by assessing the underlying affective triggers for such behavior. Using the *Risky, Impulsive, and Self-destructive Behavior Questionnaire* (Sadeh and Baskin-Sommers, 2016), we developed latent profiles of affective triggers for risky and impulsive behavior in an unselected sample of community members. Affective profiles were examined for associations with individual difference measures of personality and behavior. Additionally, we used a sample of currently incarcerated individuals to evaluate the predictive utility of affect-based profiles for substance abuse, criminal activity, and disciplinary violations while incarcerated.

## Materials and Methods

### Participants

#### Community Sample

Participants consisted of 439 male (41%) and female (59%) adults ages 18–66 (*M* = 28.7, *SD* = 12) recruited from the general community through flyers in New Haven County, Connecticut and Internet advertisements posted nationally. Individuals were eligible to participate if they were age 18 or older. Participants that were in the New Haven area came into the lab and participants who were national filled out the survey online using Qualtrics (see Supplementary Table 1 for sample characteristics). All procedures were approved by the Yale Social Science, Behavioral, and Educational Research Institutional Review Board.

#### Prison Sample

Participants consisted of 262 incarcerated male inmates ages 18–67 (*M* = 32.1, *SD* = 10.3) recruited from a maximum-security prison in Connecticut. A prescreen of institutional files and assessment materials was used to exclude individuals who had performed below the fourth-grade level on a standardized measure of reading (Wide Range Achievement Test-III; Wilkinson, 1993), who scored below 70 on a brief measure of IQ (Wechsler Adult Intelligence Scale-III; Wechsler, 1997), who had diagnoses of schizophrenia, bipolar disorder, or psychosis, not otherwise specified, or who had a history of medical problems (e.g., uncorrectable auditory or visual deficits, seizures, head injury with loss of consciousness greater than 30 min) that may have impacted their comprehension of the materials (see Supplementary Table 1 for sample characteristics). Following the prescreen, eligible inmates were called down to the office at random and were provided with written informed consent according to the procedures set forth by the Yale University Human Investigation Committee.

### Measures

#### Risky and Impulsive Self-Destructive Behavior Questionnaire (RISQ)

Participants responded to a set of 38 items that represented different risky and self-destructive behaviors. For each behavior, participants were asked to report: (a) *“How many times total have you done this in your life?,”* (b) *“How many times have you done this in the past month?,”* (c) *“How old were you the first time?,”* and (d) *“Did it ever cause you any problems, such as going to the hospital, legal trouble, problems at work, with family or friends.”* Participants were asked to rate on a 5-point Likert-type scale (0 = *“Strongly Disagree”* to 4 = *“Strongly Agree”*) how much they agreed with the following for each behavior endorsed: (e) *“I do this behavior to stop feeling upset, distressed, or overwhelmed”* and (f) *“I do this behavior to feel excitement, to get a thrill, or to feel pleasure.”* The last two questions assessing Avoidance and Approach, respectively, encompassed both the automatic action tendency and the valence motivating these behaviors. That is, the Avoidance scale included negative basic emotions (e.g., distress) and avoidance motivational impulses, whereas the Approach scale included positive basic emotions and approach motivational impulses. Though emotion and motivation are not interchangeable, these groupings across emotion and motivation categories represent two theoretically relevant conceptualizations of affective triggers for risky behaviors and there is evidence that the neural systems involved in the emotional and motivational “avoidance” versus “approach” systems are overlapping (Harmon-Jones and Harmon-Jones, 2015).

#### Measures in the Community Sample

All participants completed the following: (i) *Dimensions of Anger Reactions-5* (DAR-5; Hawthorne et al., 2006), a 5-item questionnaire that measured anger-related reactions and interference with social functioning over the past 4 weeks. The total score was calculated by summing each item (Cronbach’s alpha = 0.86); (ii) *Distress Tolerance Scale* (DTS; Simons and Gaher, 2005), a 15-item measure designed to assess the ability to withstand negative physical and psychological states. A total score was created by summing responses (Cronbach’s alpha = 0.93); (iii) *Mood and Anxiety Symptom Questionnaire – Mini* (MASQ-Mini; Clark and Watson, 1995), a 26-item questionnaire that summed symptoms of anhedonic depression (Cronbach’s alpha = 0.85), anxious arousal (Cronbach’s alpha = 0.85), and general distress (Cronbach’s alpha = 0.91) in the past week.; (iv) *Behavioral Inhibition Scale/Behavioral Activation Scale* (BIS/BAS; Carver and White, 1994), a 20-item measure that summed items related to the tendency to experience negative affect in response to threat (BIS; Cronbach’s alpha = 0.82) and to experience positive affect in response to reward (BAS; Cronbach’s alpha = 0.84); (v) *Personality Assessment Inventory* (PAI; Morey and Staff, 1991) Borderline Personality Disorder (PAI-BOR) subscale that assessed four features of Borderline Personality Disorder: affective instability, identity problems, negative relationships, and self-harm. Using a Likert scale of 0–3 (0 = false, not at all true; 1 = slightly true; 2 = mainly true; 3 = very true) participants rated each item and responses were summed (Cronbach’s alpha = 0.80); and (vi) *Michigan Assessment-Screening Test for Alcohol and Drugs* (MAST-AD; Westermeyer et al., 2004), a 25-item measure of the consequences of alcohol and drug use. Responses were given weighted scores using the standard scoring protocol (Cronbach’s alpha = 0.92).

#### Measures in the Prison Sample

Participants were assessed on the *Structural Clinical Interview for DSM-5* (SCID 5; First et al., 2015) *Substance Use Disorders* module. Additionally, official records and self-report were used to measure criminal activity and disciplinary violations within the prison.

### Data Analyses

#### Latent Profile Analysis

In order to examine whether the RISQ can be used to parse individuals based on their affective motivations to engage in risky behavior, a latent profile analysis was conducted using average scores on the Avoidance and Approach scales. A latent profile analysis allows us to identify homogenous groups identified as latent classes. Given that these RISQ scales were correlated with lifetime engagement in risky behaviors (*r’*s = 0.25 to 0.36, *p*-values <0.001), we first residualized the average ratings on the affective scales by total lifetime engagement in risky behavior before submitting them to the latent profile analysis. We evaluated five latent profile models (2- through 6-class models). The relative fit of the models was compared using the Bayesian Information Criterion (smaller values indicate better model fit; Schwartz, 1978), entropy (higher values indicate better ability to classify participants and discriminate among classes; Ramaswamy et al., 1993), and the Lo–Mendell–Rubin-adjusted likelihood ratio test (Lo et al., 2001) where a significant *p*-value indicates that a model with *k* number of classes is preferred over a model with *k*-1 classes. Classes containing less than 5% of the sample were rejected due to concerns that they may not be representative (Nylund et al., 2007). Latent profile analyses were performed in Mplus 7.11 using the maximum likelihood robust estimator (Muthén and Muthén, 2013).

The profiles generated from the community sample were applied to the prison sample, by generating class estimates from the community latent profile analysis and fitting those estimates to participants in the prison sample. The goal of this approach was to examine whether general latent classes could provide predictive utility in a different (i.e., prison) sample^1^.

#### Conditional Probability Correlations

Comparison of latent profiles was examined by correlating the probability of membership in each class with external variables using Pearson correlations. Differences in the magnitude of pairs of associations between external variables and the probability of membership in different latent classes were evaluated using a test of dependent correlations (Lee and Preacher, 2013, available at http://quantpsy.org). Conditional probabilities were used in order to reduce the impact of unbalanced sample sizes across latent classes. All tests were completed using SPSS version 23.0 (IBM SPSS, Chicago, IL, United States).

## Results

### Latent Profile Analyses in the Community Sample

Model fit for solutions with 2–6 latent classes were examined (see Table 1). The Lo–Mendell–Rubin-adjusted LRT indicated that models with 2, 4, and 6 classes showed improved fit over those with one fewer class. Of these, the six-class solution was rejected because the best log-likelihood value was not replicated and two of the classes contained less than 5% of the sample. The four-class solution was ultimately selected as the best fitting model because it showed better discrimination among the classes (higher entropy value at 0.68) and a lower BIC value (2434.78) than the two-class solution. Individuals were then classified according to their most likely class membership. As illustrated in Supplementary Figure 1, the first class was characterized by low ratings on both Avoidance and Approach scales (class 1 [Low/Low]; 24% of the sample), the second class was characterized by average Avoidance and average Approach ratings (class 2 [Average]; 48% of the sample), the third class was marked by high ratings on Avoidance and average Approach (class 3 [High Avoid]; 20% of the sample), and the fourth class reflected low ratings for Avoidance and high ratings for Approach (class 4 [High App]; 8% of the sample).

**Table 1:** Model fit of the latent profile analysis in a community sample.

No. latent classes	Log-likelihood	BIC	Adjusted BIC	Adjusted LMR LRT *p*	Bootstrap LRT *p*	Entropy
2-Class	-1240.96	2478.61	2456.40	0.000	<0.001	0.60
3-Class	-1218.08	2481.56	2449.83	0.312	<0.001	0.62
4-Class	-1210.37	2476.03	2434.78	0.034	<0.001	0.68
5-Class	-1207.65	2479.72	2428.95	0.557	<0.001	0.72
6-Class	-1191.35	2467.06	2406.76	0.003	<0.001	0.81

*BIC, Bayesian Information Criterion; LMRA-LRT p, Lo–Mendell–Rubin-adjusted Likelihood Ratio Test p-value; LRT, likelihood ratio test.*

**FIGURE 1:**
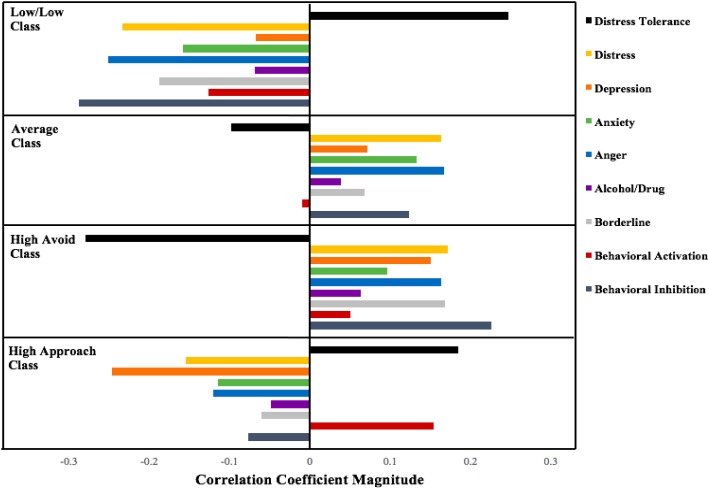
Probability of class membership correlations with external variables by RISQ affective profiles in a community sample.

To explore the external correlates of these affective profiles, we examined how they differed on measures of motivation, distress tolerance, borderline personality features, mood and anxiety symptoms, and alcohol/drug use problems. Results of these analyses are presented in Table 2 and Figure 1. Probability of membership in the class characterized by low ratings on both of the RISQ affect scales (Low/Low) was associated with greater resilience to distress, lower mood and anxiety symptoms, fewer borderline personality features, and less behavioral inhibition and activation than membership in the other classes. Probability of membership in the class marked by distinctly high ratings on RISQ Avoidance motivation (High Avoid), in contrast, was associated with significantly lower distress tolerance than membership in the other three classes. Furthermore, membership in the High Avoid class was related to higher ratings of behavioral inhibition, mood and anxiety symptoms, borderline personality features, and alcohol/drug use than membership in the classes with low ratings on RISQ Avoidance (classes 1 and 4). In contrast, the probability of membership in the class characterized by high ratings selectively on RISQ Approach motivation (High Approach) was associated with greater behavioral activation (BAS) and lower anhedonic depression symptoms than the probability of membership in the classes marked by low to average ratings on RISQ Approach motivation (classes 1 and 2).

**Table 2:** External correlates of RISQ affective profiles in a community sample.

	C. Prob. 1 (Low/Low)	C. Prob. 2 (Average)	C. Prob. 3 (High Avoid)	C. Prob. 4 (High App)	Correlation comparison
	A	B	C	D	
Behavioral inhibition system	-0.287	0.123	0.226	-0.077	A vs. B, A vs. C, A vs. D, B vs. D, C vs. D
Behavioral activation system	-0.126	-0.01	0.051	0.154	A vs. B, A vs. C, A vs. D, B vs. D
Borderline features	-0.187	0.068	0.168	-0.06	A vs. B, A vs. C, A vs. D, B vs. D, C vs. D
Distress tolerance scale	0.247	-0.098	-0.279	0.185	A vs. B, A vs. C, B vs. C, B vs. D, C vs. D
Dimensions of anger reactions	-0.251	0.167	0.164	-0.12	A vs. B, A vs. C, A vs. D, B vs. D, C vs. D
MASQ general distress	-0.233	0.163	0.172	-0.154	A vs. B, A vs. C, B vs. D, C vs. D
MASQ anxious arousal	-0.158	0.133	0.096	-0.114	A vs. B, A vs. C, B vs. D, C vs. D
MASQ anhedonic depression	-0.067	0.072	0.15	-0.246	A vs. B, A vs. C, A vs. D, B vs. D, C vs. D
MAST/AD	-0.069	0.039	0.063	-0.049	A vs. C, C vs. D

*Low/Low (A), scored lowest on both RISQ affect scales. Average (B), average scores on both RISQ affect scales. High Avoid (C), high Avoidance group. High App (D), high Approach group. Borderline personality features scores derived from consolidated *z*-score totals reflecting the Personality Assessment Inventory (PAI)-BOR and the SCID-IV Borderline personality disorder symptom counts. MASQ, Mood and Anxiety Symptom Questionnaire – Mini. MAST/AD, Michigan Assessment-Screening Test/Alcohol-Drug. Evaluation of the statistical significance of the difference in the magnitude of pairs of correlations was conducted using *t*-tests to compare dependent correlations. C. Prob., conditional probability of class membership.*

### Predictive Affect Profiles in the Prison Sample

Using the latent profile structure extracted from the community sample, we applied the four-class solution to the prison sample. Class 1 (Low/Low) characterized 30% of the sample, class 2 (Average) characterized 47% of the sample, class 3 (High Avoid) characterized 16% of the sample, and class 4 (High App) characterized 7% of the sample.

**FIGURE 2:**
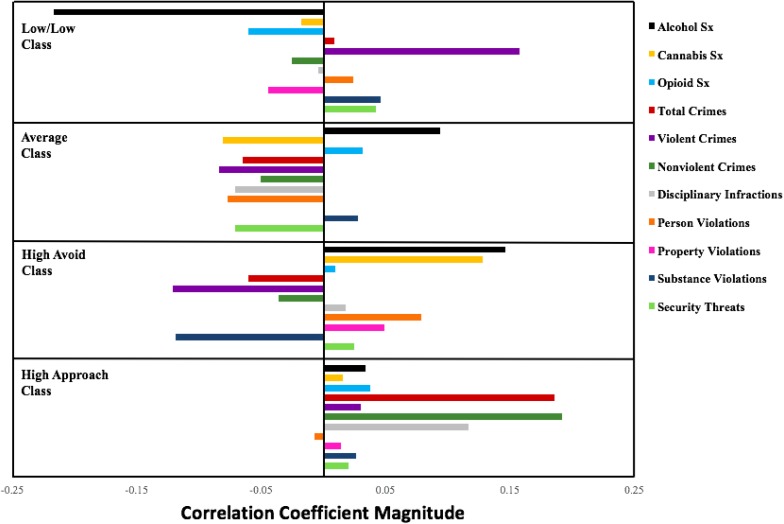
Probability of class membership correlations with external variables by RISQ affective profiles in a prison sample.

In terms of substance use, the probability of membership in the class marked by selectively high ratings on RISQ Avoidance (class 3) was associated with higher prior alcohol use symptoms and cannabis use symptoms than the classes characterized by low and average scores on the RISQ affect scales (classes 1 and 2, respectively). Next, in terms of criminal activity, probability of membership in the High Approach class was associated with higher total number of crimes and non-violent crimes than membership in the other classes. Probability of membership in the class marked by low ratings on both of the RISQ affect scales (class 1) was associated with higher total number of violent crimes than membership in class 2 or 3, though this relationship was largely driven by higher numbers of sex crimes in class 1. Finally, in terms of disciplinary violations within prison, the probability of membership in the High Approach class was associated with higher total number of disciplinary violations than membership in class 2. Probability of membership in the High Avoid class was associated with higher number of violations against persons than membership in class 2 but lower number of substance use violations than membership in classes with low and average ratings on the RISQ affect scales (classes 1 and 2). Further, probability of class membership in the Average class was associated with fewer total days in segregation as punishment for substance use violations than membership in classes marked by high avoid and approach, respectively (classes 3 and 4) (see Table 3 and Figure 2).

**Table 3:** External correlates of RISQ affective profiles in a prison sample.

	C. Prob. 1 (Low/Low)	C. Prob. 2 (Average)	C. Prob. 3 (High Avoid)	C. Prob. 4 (High App)	Correlation comparison
	A	B	C	D	
Alcohol use symptoms count	-0.217	0.094	0.146	0.034	A vs. B, A vs. C, A vs. D
Cannabis use symptoms count	-0.018	-0.081	0.128	0.016	B vs. C
Opioid use symptoms count	-0.060	0.032	0.010	0.038	
Total number of crimes	0.009	-0.065	-0.060	0.186	A vs. D, B vs. D, C vs. D
Total number of violent crimes	0.158	-0.084	-0.121	0.030	A vs. B, A vs. C, C vs. D
Total number of non-violent crimes	-0.025	-0.050	-0.036	0.192	A vs. D, B vs. D, C vs. D
Total number of disciplinary violations	-0.004	-0.071	0.018	0.117	B vs. D
Total number of violations against persons	0.024	-0.077	0.079	-0.007	B vs. C
Total days in segregation	-0.176	0.213	-0.031	-0.039	
Total number of violations against property	-0.044	0.001	0.049	0.014	
Total days in segregation		1.000	-1.000		
Total number of substance use violations	0.046	0.028	-0.119	0.026	A vs. C, B vs. C
Total days in segregation		-0.803	0.448	0.564	B vs. C, B vs. D
Total number of threats to security	0.042	-0.071	0.025	0.020	
Total days in segregation	-0.153	0.212	-0.089		

*Total days in segregation, total days segregated or in solitary confinement as a result of disciplinary infraction. Evaluation of the statistical significance of the difference in the magnitude of pairs of correlations was conducted using t-tests to compare dependent correlations. C. Prob., conditional probability of class membership.*

## Discussion

Risky and impulsive behavior is ubiquitous, it spans age, race, gender, and socioeconomic categories. Considering the underlying factors that influence why an individual engages in this type of behavior is essential. Throughout the years, numerous theoretical conceptualizations of risky and impulsive behavior have been proffered, but most fundamentally they all centered on the broad tenets of avoidance and approach. James (1890) discussed pain as a “tremendous inhibitor” of behavior and pleasure as a “tremendous reinforcer,” mapping these triggers onto “inhibitory” and “impulsive” tendencies (p. 550). Eysenck (1967) suggested that introverts have a high baseline level of cortical arousal and, therefore, typically avoid additional stimulation (“stimulus shyness”), whereas extraverts have a low baseline level of arousal and, therefore, typically seek additional stimulation (“stimulus hunger”). Gray (1982) highlighted the balance between behavioral inhibition and activation systems as being important for maintaining controlled behavior. And, more recently, theories stressed individual differences in distress tolerance or pleasure seeking as important in the development and maintenance of risky and impulsive behaviors (Zuckerman and Kuhlman, 2000; Leyro et al., 2010). Despite the abundance of evidence implicating avoidance and approach tendencies in risky behaviors, few assessments directly measure these tendencies. Furthermore, although studies have examined subtypes of individuals who engage in specific types of risky behavior (e.g., gambling typologies; Stewart et al., 2008), this is the first study to examine affect-based subtypes across a diverse range of risk behaviors.

In the present study, four distinct profiles characterized the affective triggers for risky behavior. Not surprisingly, there was a subset of people who engaged in risky behaviors primarily because of avoidance tendencies (e.g., negative affect, distress) and another subset who engaged in risky behaviors primarily because of approach tendencies (e.g., pleasure, thrill seeking). Moreover, some individuals tended to report risky behaviors in the context of low or average levels of both avoidance/approach tendencies.

Individuals who were primarily motivated by avoidance reported higher levels of behavioral inhibition and symptomatology related to mood and anxiety disorders, and lower ability to tolerate distress. This profile among incarcerated individuals was predictive of alcohol and marijuana use disorders and disciplinary violations (assault, threats, fighting, sex assault, riots) against people within the prison. It is possible that this profile captures individuals who use substances to self-mediate, who struggle to tolerate unpleasant states, and who have difficulty disengaging from risky behavior in the face (e.g., interpersonal interactions) of psychological or physical distress (Baker et al., 2004; see Baskin-Sommers and Hearon, 2015 for review; Nelson et al., 2008; Leyro et al., 2010; Nock, 2010). By contrast, individuals with largely approach motivation for risky behavior reported relatively strong abilities to tolerate distress, lower levels of mood symptomatology, and higher levels of behavioral activation. Within the prison, these individuals represented the highest risk group in terms of previous criminal activity (e.g., total number of crimes) and disciplinary violations within the prison. Positive affect encourages action tendencies, such as tenacious goal-directed behavior, but often at the expense of attentional breadth and incorporation of information that conflicts with obtaining goals (Harmon-Jones and Harmon-Jones, 2015). Therefore, individuals motivated by high approach tendencies may continually pursue rewards or feel amped up by risky situations despite the serious potential consequences (e.g., Holub et al., 2005; Zapolski et al., 2009; Leonard, 2010; Derefinko et al., 2011; Willmott and Ioannou, 2017). Finally, the Low/Low and Average groups may represent individuals who engage in risky behavior due to generally low and average levels of arousal or other triggers aside from affective motivations, respectively (Fung et al., 2005). Notably, these individuals do engage in risky behaviors, but affective triggers do not seem to be a discriminatory underlying process for their behavior. For example, in the prison sample, it was the Low/Low group that had the highest levels of violent crimes, particularly sex offenses. Some theories about individuals who engage in violent behavior posit that these individuals are motivated by blunted affect, rather than higher affective reactivity (see Raine, 2013 for review). Continuing to understand the range of triggers and associated behavioral profiles for risky and impulsive behavior is important.

Together, these four profiles may represent the influence of levels of affect on behavior along a continuum. At the low end and in the middle, affective tendencies may not dissociate or may be non-specific predictors of behavior. However, at the high end of arousal, avoidance or approach affective tendencies become predominant and a primary affective trigger emerges to best characterize risky behavior within an individual. The emergence of distinct affective profiles highlights the importance of assessing both behavioral engagement across high-risk behaviors and people’s differing emotions and motivations for engaging in them. One advantage of this approach is that it may have greater transdiagnostic relevance for understanding affective profiles for risky behavior than previous research on category-specific subtypes. Further, examining affective triggers for a range of risky behaviors has high ecological validity, given that individuals who engage in one type of risky behavior are at higher risk for other types of risky and impulsive behaviors (Thomsen et al., 2011). Thus, this approach is well positioned to identify shared etiological mechanisms across these frequently co-occurring problem behaviors.

Several limitations should be noted. First, although our data indicated a 4-class solution best fit the data, it is entirely possible that other profiles exist, and further exploration of such subtypes should be examined in future research on the *RISQ*. Second, the sample sizes in the community and prison samples were moderate in size. It is possible that larger samples would produce different class solutions that better separate individuals (i.e., higher entropy; Ramaswamy et al., 1993; Tein et al., 2013). Third, measurement of affect triggers was based solely on the individual’s self-report, and this report was temporally quite separated from engagement in the behavior. It is possible that some individuals are unaware of the reasons why they engage in risky and impulsive behavior, thereby affecting the reliability of their ratings. Future research should combine objective and subjective measures of affective triggers and consider employing *in vivo* assessments to evaluate affective states prior to and immediately post behavior. Despite these limitations, the study also has several strengths, including use of latent profile analysis to identify empirically derived affective subtypes, recruitment of a clinically relevant sample of incarcerated adults who show elevated rates of risky and impulsive behaviors, and examination of a range of external correlates spanning normative (e.g., behavioral activation) and pathological outcomes (violent crimes).

The present results contribute a strong empirical model that future research on the affective triggers for risky and impulsive behavior can build upon. Given that risky and impulsive behaviors are transdiagnostic features of many psychiatric diagnoses and social problems (Berg et al., 2015), research on the etiology of these harmful behaviors may benefit from person-centered approaches that move away from diagnostic categories and toward classification systems based on shared underlying mechanisms (e.g., Cuthbert, 2014). The current results provide empirical support for this conceptualization and suggest that classifying individuals based on differences in the valence of the affect that triggers their behaviors (approach vs. avoidance) and level of arousal they feel when engaging in these behaviors (low, average, or high) translates into clinically distinct groups with unique personality, mental health, and behavioral profiles. The assessment of affective risk factors and behavioral propensities is important for developing comprehensive risk assessments that more precisely characterize underlying motives along with behavior. The application of increased precision in assessment can be translated into more effective “needed assessments” and selection of treatment programs (Bonta and Andrews, 2007). An important next step in this line of research is to closely examine the mechanistic processes that differentiate these individuals across neurobiological, cognitive, and affective levels analyses, information that can then be translated into targeted, and potentially more personalized, assessment and interventions aimed at reducing engagement in risky behaviors. Together, targeted and effective identification, prevention, and intervention strategies that move away from broad outcome-based classifications and move toward an integrative understanding of affect-behavior associations is important for addressing the impact of risky, impulsive, and self-destructive behavior. Thus, by showing for the first time that it is possible to distinguish individuals based on affective profiles for a spectrum of risky behaviors, this study contributes a novel classification model that has varied future applications for both research and implementation in clinical and forensic settings.

## Author Contributions

AB-S and NS collected the data. EK, NS, and AB-S conducted the analyses and wrote the manuscript.

## Conflict of Interest Statement

The authors declare that the research was conducted in the absence of any commercial or financial relationships that could be construed as a potential conflict of interest.

## Footnotes

1 Typically, with latent profile analyses, larger samples are needed to generate good fit statistics. The prison sample was on the small end for these types of analyses. This partially motivated the choice to apply latent profiles generated by a larger sample to the prison sample. However, for the sake of completeness we did run the latent profile analysis in the prison sample. Just like the community sample, four latent classes provided a strong fit (entropy = 0.68, BIC = 1293.61). Combined, these fit statistics were better than solutions generated two (entropy = 0.64, BIC = 1298.21), three (entropy = 0.57, BIC = 1297.11), and six classes (entropy = 0.73, BIC = 1308.76) (see Supplementary Table 2). These statistics were similar to the five-class solution (entropy = 0.75, BIC = 1294.76), but in the five class solution too few participants (3%) were in the fifth class.
