# Supplementary material for: A Latent Profile Analysis of Affective Triggers for Risky and Impulsive Behavior
Source: Front Psychol. 2019 Jan 4;9:2651. doi: 10.3389/fpsyg.2018.02651 (PMC6328440; doi:10.3389/fpsyg.2018.02651)
Supplement: Supplementary file 1 [file Table_1.docx]

**A Latent Profile Analysis of Affective Triggers for Risky and Impulsive Behavior: Supplemental Materials**

Supplemental Table 1. *Participant* *Demographics by Sample*

|  | Community Sample | Prison Sample |
| --- | --- | --- |
| N | 439 | 262 |
| Age *(M/SD)* | 28.7/ 12.0 | 32.1/ 10.3 |
| Female Gender (*N/ %*) | 259/ 59.0 | 0/ 0.0 |
| Race (*N/ %*) |  |  |
| White | 269/ 61.3 | 110/ 42.0 |
| African-American | 79/ 18.0 | 142/ 54.2 |
| Asian | 49/ 11.2 | 1/ 0.4 |
| Other | 2/ 0.4 | 7/ 2.7 |
| Mixed Race | 40/ 9.1 | 2/ 0.8 |
| Ethnicity (*N/ %*) |  |  |
| Hispanic | 49/ 11.1 | 45/ 17.2 |
| Not Hispanic | 376/ 85.5 | 215/ 82.1 |
| Unknown | 14/ 3.2 | 0/ 0.0 |
| Education (*N/ %*) |  |  |
| ≤12 Years | 26/ 5.9 | 167/ 20.8 |
| HS Diploma/GED | 70/ 15.9 | 74/ 28.2 |
| Some College/Bachelor’s Degree | 235/ 53.5 | 16/ 6.1 |
| Graduate Work or Degree | 108/ 24.6 | 2/ 0.8 |
| Employment (*N/ %*) |  |  |
| Full Time | 47/ 10.7 | -- |
| Part Time | 54/ 12.3 | -- |
| Unemployed | 65/ 14.8 | -- |
| Retired/ Disability | 15/ 3.4 | -- |
| Full Time Student | 258/ 58.6 | -- |

*Note*. Other ethnicity includes Native American, American Indian, Alaskan Native, or Native Hawaiian Pacific Islander. HS = high school; GED = General Equivalency Diploma.

Supplemental Table 2. *Model Fit of the Latent Profile Analysis in a Prison Sample*

| No. latent classes | Log-likelihood | BIC | Adjusted BIC | Adjusted LMR LRT *p* | Bootstrap  LRT *p* | Entropy |
| --- | --- | --- | --- | --- | --- | --- |
| 2-Class | -630.04 | 1298.21 | 1276.02 | 0.006 | <0.001 | 0.64 |
| 3-Class | -621.32 | 1297.11 | 1265.41 | 0.232 | <0.001 | 0.57 |
| 4-Class | -611.40 | 1293.61 | 1252.40 | 0.343 | <0.001 | 0.68 |
| 5-Class | -603.80 | 1294.76 | 1244.05 | 0.022 | <0.001 | 0.75 |
| 6-Class | -602.64 | 1308.76 | 1248.54 | 0.572 | 1.000 | 0.73 |

*Note*. BIC = Bayesian Information Criterion. LMRA-LRT *p* = Lo-Mendell-Rubin–adjusted Likelihood Ratio Test *p*-value; LRT = Likelihood Ratio Test.

Supplemental Figure 1. *Average Approach and Avoidance Motivation Ratings by RISQ Affective Profiles*

*Note:* Letters denote significant differences in average motivation ratings for approach motivation or avoidance motivation using one-way ANOVA with Bonferroni correction.
